# Tracheobronchial pen cap and pen nib foreign body aspiration in 32 children: clinical features, CT findings, and bronchoscopic management

**DOI:** 10.1186/s12887-026-07395-0

**Published:** 2026-07-30

**Authors:** Zhao Cheng, Qi Cheng, Ying Yang, Shuhua Wu

**Affiliations:** Department of Otorhinolaryngology, Anhui Hospital of Children’s Hospital of Fudan University, Anhui Maternity and Child Health Hospital, Anhui Provincial Children’s Hospital, Hefei, Anhui Province 230022 China

**Keywords:** Child, Foreign body aspiration, Tracheobronchial foreign body, Bronchoscopy, Computed tomography

## Abstract

**Background:**

Tracheobronchial foreign body aspiration is a common pediatric emergency. In recent years, aspiration of stationery-related objects such as pen caps and pen nibs has been increasingly encountered in school-aged children. These objects possess unique structural characteristics that may influence clinical presentation and management.

**Methods:**

We conducted a retrospective analysis of 32 pediatric patients with tracheobronchial foreign body aspiration caused by pen caps or pen nibs treated between January 2017 and December 2025. Clinical features, imaging findings, foreign body characteristics, bronchoscopic management, and outcomes were analyzed. The relationship between provision of a foreign body history and disease duration was evaluated.

**Results:**

All 32 patients were school-aged boys (mean age, 8.6 years). The most common symptoms were cough and wheezing. Chest CT was performed in 31 patients (96.9%) and identified/localized the foreign body in all examined cases; however, this retrospective study did not evaluate whether CT independently changed management or outcomes. Foreign bodies were most frequently located in the right main bronchus. All cases were successfully managed using rigid bronchoscopy, with flexible bronchoscopy used as an adjunct in one case. Patients who did not provide a clear or timely history of foreign body aspiration had a significantly longer disease course compared with those with a definite history (*P* < 0.001). No life-threatening complications occurred; however, clinically relevant events such as transient hemoptysis, airway edema, and pneumothorax were observed in individual cases.

**Conclusions:**

Stationery-related tracheobronchial foreign body aspiration predominantly affects school-aged boys and presents unique diagnostic challenges. Delayed or absent reporting of aspiration history is associated with prolonged disease duration. CT may be considered as a selective adjunct for localization and procedural planning in clinically stable children, particularly when the history is unclear or presentation is atypical, while rigid bronchoscopy remains the primary diagnostic and therapeutic modality.

**Supplementary Information:**

The online version contains supplementary material available at https://doi.org/10.1186/s12887-026-07395-0.

## Introduction

Tracheobronchial foreign body aspiration is a common and potentially life-threatening emergency in children. Delayed diagnosis or inappropriate management may result in serious complications, including recurrent pneumonia, atelectasis, bronchiectasis, and even death [1, 2]. However, early clinical manifestations are often nonspecific and may mimic common respiratory infections, which contributes to missed or delayed diagnosis [3].

The types of aspirated foreign bodies vary according to age, environment, and daily activities. Food-related foreign bodies are most frequently encountered in infants and toddlers, whereas non-food objects are increasingly reported in school-aged children [4]. Among these, stationery-related items such as pen caps and pen nibs represent a distinctive subgroup. These objects are commonly used in school settings and are often habitually held in the mouth during writing or play, increasing the risk of accidental aspiration.

Pen caps and pen nibs possess unique physical characteristics that may influence both clinical presentation and bronchoscopic management. Their hollow or tubular structures may allow partial airflow after aspiration, potentially delaying the onset of severe symptoms and contributing to delayed recognition [5]. In addition, their smooth surfaces and, in some cases, sharp edges may facilitate distal migration and increase technical difficulty during extraction.

Imaging plays an important role in the evaluation of suspected airway foreign bodies. Chest radiography may demonstrate indirect findings such as obstructive emphysema or atelectasis but often fails to directly visualize radiolucent objects [6]. Computed tomography (CT) may assist in identifying and localizing foreign bodies and in assessing secondary pulmonary changes, thereby supporting procedural planning, although its role should be considered in the clinical context [7].

Bronchoscopy remains the gold standard for both diagnosis and treatment of tracheobronchial foreign bodies in children. Rigid bronchoscopy provides optimal airway control and allows the use of various retrieval instruments, whereas flexible bronchoscopy may serve as a useful adjunct in selected cases, particularly when visualization or access is challenging [8].

Although previous studies have described pediatric foreign body aspiration in general, limited data are available focusing specifically on stationery-related foreign bodies such as pen caps and pen nibs. In particular, the potential impact of their structural characteristics on clinical presentation and the role of delayed or absent aspiration history have not been well characterized.

In this study, we retrospectively analyzed 32 pediatric cases of tracheobronchial foreign body aspiration caused by pen caps and pen nibs. Rather than focusing on the general effectiveness of rigid bronchoscopy, which has been well established, this study aimed to characterize pen caps and pen nibs as a clinically relevant subgroup of hollow or tubular stationery-related foreign bodies in school-aged children. We specifically focused on their clinical presentation, imaging features, association with delayed recognition, and bronchoscopic retrieval considerations.

## Methods

### Study design and setting

This retrospective observational study included pediatric patients with tracheobronchial foreign body aspiration caused by pen caps or pen nibs who were treated at a tertiary children’s hospital between January 2017 and December 2025. The study was conducted in accordance with institutional policies for retrospective clinical research.

### Participants

Children aged ≤ 14 years who underwent bronchoscopic removal of tracheobronchial foreign bodies identified as pen caps or pen nibs were eligible for inclusion. Diagnosis was confirmed by imaging and/or bronchoscopy. Patients with food-related foreign bodies or non-stationery objects were excluded.

### Data collection

Clinical data were extracted from electronic medical records. Variables collected included age, sex, presenting symptoms, provision of foreign body aspiration history, symptom duration, imaging findings, foreign body type (pen cap or pen nib), anatomical location, bronchoscopic technique, perioperative events, and clinical outcomes (Table 1). Symptom duration was defined as the interval between symptom onset or suspected aspiration and bronchoscopic removal.

**Table 1:** Baseline demographic and clinical characteristics (*n* = 32)

Characteristic	Value
Age, years	8.6 ± 2.0 (range 4–13)
Sex	Male, 32 (100%)
Provided history of foreign body aspiration	25 (78.1%)
No clear or timely history	7 (21.9%)
Symptom duration, hours	Median 12 (range 3–480)
Presentation ≤ 24 h	23 (71.9%)
Presentation > 24 h	9 (28.1%)
Cough/wheezing	30 (93.8%)
Cough/wheezing with dyspnea	2 (6.2%)

### Imaging evaluation

Children with suspected tracheobronchial foreign body aspiration were initially evaluated according to aspiration history, presenting symptoms, physical examination, oxygenation status, and signs of airway compromise. Bronchoscopy was prioritized and was not delayed for CT in children with unstable vital signs, severe respiratory distress, or a definite aspiration history with significant airway symptoms. In clinically stable children, chest CT was considered when the aspiration history was unclear or delayed, when symptoms were atypical, when chest radiography, if performed, was negative or inconclusive, or when additional information regarding foreign body location, morphology, or secondary pulmonary changes was needed before bronchoscopy.

In this cohort, CT was performed in 31 of 32 patients, while one patient proceeded directly to bronchoscopy without preoperative CT because of a clear aspiration history and significant respiratory symptoms. CT findings were used to support foreign body localization and preoperative planning. However, CT was not considered mandatory for all suspected cases, and the retrospective design of this study did not allow standardized assessment of whether CT findings changed management decisions or clinical outcomes (Table 2).

**Table 2:** Imaging findings and anatomical distribution

Variable	*n* (%)
Chest CT performed	31 (96.9%)
Location of foreign body	
– Right main bronchus	23 (71.9%)
– Left main bronchus	8 (25.0%)
– Trachea	1 (3.1%)

### Bronchoscopic procedure

All procedures were performed under general anesthesia by experienced pediatric bronchoscopists. Rigid bronchoscopy was used as the primary technique for foreign body removal in all cases. Flexible bronchoscopy was used as an adjunct in selected cases to assist with visualization or retrieval.

Rigid bronchoscopes (Karl Storz) of appropriate sizes were selected according to patient age and airway diameter. Foreign body retrieval was performed using instruments including peanut forceps, alligator forceps, and biopsy forceps. Flexible bronchoscopy (Olympus P290 series) could be introduced either through the rigid bronchoscope or via an endotracheal tube, depending on procedural requirements.

Advanced retrieval devices such as baskets or balloon catheters were not routinely available in our center during the study period. Given the hollow structure of pen caps and pen nibs, intraluminal grasping using forceps was considered a stable and effective technique.

### Anesthesia and perioperative management

Procedures were performed under intravenous or combined intravenous–inhalation general anesthesia. Anesthetic agents included sevoflurane, propofol, vecuronium, and fentanyl. Ventilation was provided via the rigid bronchoscope with controlled ventilation, and endotracheal intubation was used when clinically indicated.

Intraoperative monitoring included pulse oxygen saturation and electrocardiography. Postoperatively, patients were monitored in the post-anesthesia care unit, with intensive care support provided when necessary.

### Postoperative follow-up

All patients were followed up for 2 weeks to 3 months via telephone and online communication. No obvious postoperative complications were observed, and all patients achieved complete recovery.

### Outcome definitions

Technical success was defined as complete removal of the foreign body without the need for surgical intervention. Perioperative events were categorized as severe, including mortality, massive hemorrhage, or airway perforation, or non-severe, including transient hemoptysis, airway edema, or pneumothorax requiring intervention. Procedural characteristics and outcomes are summarized in Table 3. Successful removal was defined as complete extraction of the foreign body without the need for surgical intervention.

**Table 3:** Bronchoscopic management and outcomes (*n* = 32)

Outcome	Value
Rigid bronchoscopy	32 (100%)
Flexible bronchoscopy adjunct	1 case
Successful removal	32 (100%)
Surgical intervention required	0
Severe complications	0
Non-severe complications	Present (hemoptysis, airway edema, pneumothorax)

### Statistical analysis

Continuous variables were summarized as means with standard deviations or medians with ranges, as appropriate. Categorical variables were expressed as frequencies and percentages. Given the small sample size and non-normal distribution of variables, nonparametric tests were used. The Mann–Whitney U test was applied to compare symptom duration between groups based on the presence or absence of a reported foreign body history. Correlation analysis was performed using Spearman’s rank correlation coefficient. A two-sided P value < 0.05 was considered statistically significant.

## Results

### Patient characteristics

A total of 32 children with tracheobronchial foreign bodies caused by pen caps or pen nibs were included in the analysis. All patients were male, with ages ranging from 4 to 13 years (mean age, 8.6 ± 2.0 years). Baseline demographic and clinical characteristics are summarized in Table 1.

The duration of symptoms ranged from 3 to 480 h, with a median duration of 12 h. Twenty-three patients (71.9%) presented within 24 h of symptom onset, whereas nine patients (28.1%) had symptom durations exceeding 24 h. Seven patients (21.9%) did not provide a clear or timely history of foreign body aspiration.

The most common presenting symptoms were cough and wheezing, observed in 30 patients (93.8%), while two patients (6.2%) presented with cough or wheezing accompanied by dyspnea.

### Imaging findings

**Fig. 1:**
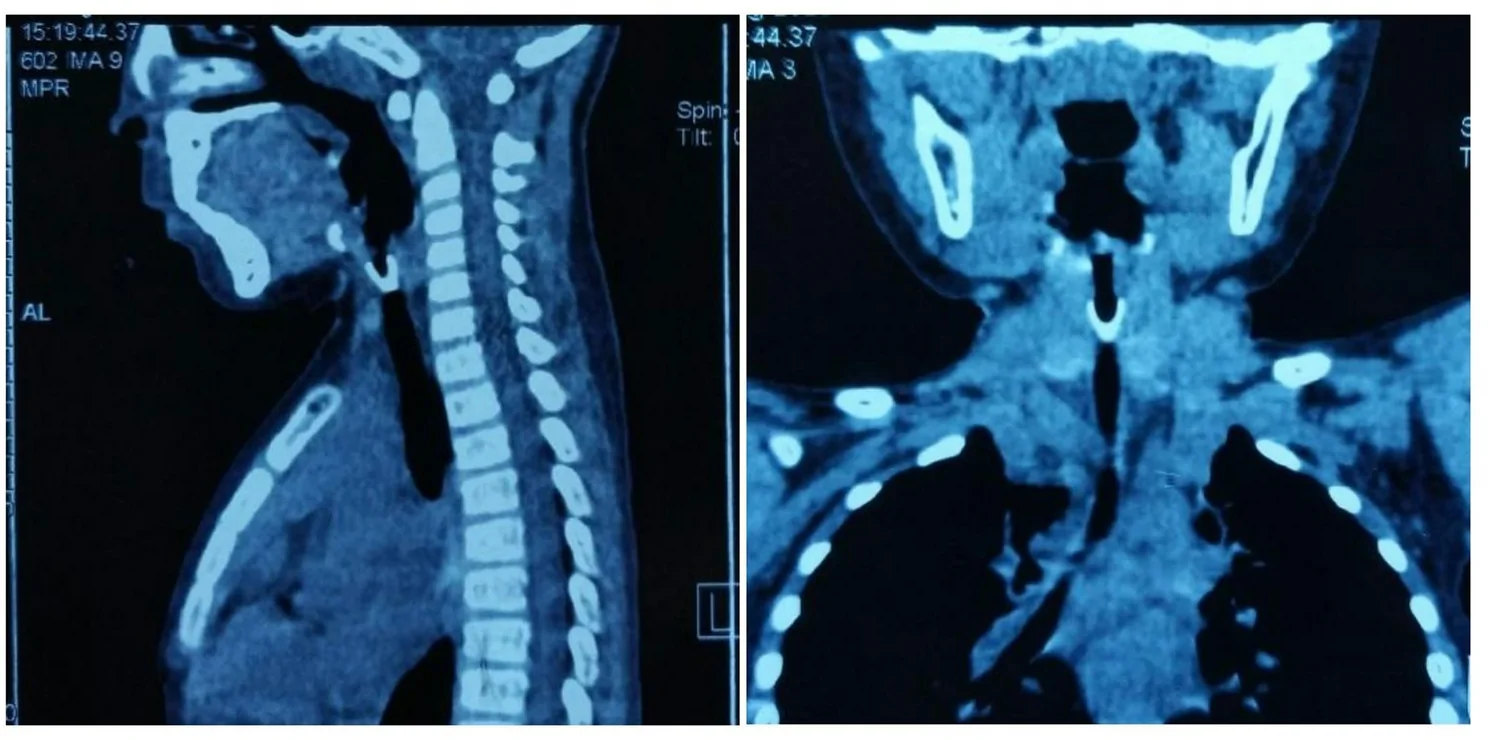
Representative CT images showing a tracheobronchial foreign body

Preoperative imaging findings are summarized in Table 2. Chest CT was performed in 31 patients (96.9%), and the foreign body was identified and localized in all patients who underwent CT (31/31, 100.0%). One patient with a clear aspiration history and significant respiratory symptoms proceeded directly to bronchoscopy without preoperative CT. CT also showed secondary pulmonary changes, including obstructive emphysema, atelectasis, and inflammatory changes. Because all patients ultimately underwent bronchoscopy, this study could not determine whether CT independently changed the decision to perform bronchoscopy, instrument selection, bronchoscopic approach, or clinical outcomes. Representative CT images demonstrating foreign body location are shown in Fig. 1.

### Foreign body type and anatomical distribution

Among the 32 cases, 18 foreign bodies were pen nibs and 14 were pen caps. Foreign bodies were most commonly located in the right main bronchus (23 cases, 71.9%), followed by the left main bronchus (8 cases, 25.0%), with one case (3.1%) located in the trachea (Table 2).

No statistically significant association was observed between foreign body type and anatomical location (*P* = 0.589; Table 4). Correlation analysis showed no significant relationship between age and symptom duration (*P* = 0.928; Table 4).

### Association between history and disease duration

Patients who did not provide a clear or timely history of foreign body aspiration had a significantly longer disease duration compared with those with a definite history (*P* < 0.001; Table 4).

**Table 4:** Statistical associations

Comparison	Test	*P* value
Foreign body type vs. location	Chi-square	0.589
Age vs. symptom duration	Spearman correlation	0.928
History vs. disease duration	Mann–Whitney U	< 0.001

### Bronchoscopic management and outcomes

All patients underwent bronchoscopic removal under general anesthesia. Rigid bronchoscopy was used as the primary technique in all cases, with flexible bronchoscopy used as an adjunct in one case (Table 3).

Technical success was achieved in all patients (100%), and no surgical intervention was required. No life-threatening complications such as mortality, massive hemorrhage, or airway perforation were observed. However, clinically relevant perioperative events did occur, including transient hemoptysis, airway edema requiring short-term intensive care monitoring, and pneumothorax requiring intervention in an individual case.

Representative cases illustrating technical challenges, including failed initial extraction, prolonged operative time, and management of complications, are summarized in the Supplementary Material.

## Discussion

In this retrospective study of 32 pediatric patients with tracheobronchial foreign body aspiration caused by pen caps and pen nibs, we identified several clinically relevant findings. These foreign bodies predominantly occurred in school-aged boys, were most frequently located in the right main bronchus, and were successfully managed using bronchoscopic techniques. More importantly, our findings highlight the potential impact of foreign body characteristics and delayed or absent aspiration history on clinical presentation and disease course.

Stationery-related foreign bodies represent a distinct subgroup compared with commonly reported food-related objects in younger children [4]. In our cohort, all patients were school-aged boys, which is consistent with previously reported male predominance in pediatric foreign body aspiration [8]. However, this observation should be interpreted with caution given the relatively small sample size, and sampling variability cannot be excluded. Behavioral factors, such as the habit of holding pen caps or pen nibs in the mouth during school activities, may contribute to this pattern.

A notable feature of pen caps and pen nibs is their hollow or tubular structure. This characteristic may allow partial airflow after aspiration, potentially resulting in milder or delayed symptoms compared with complete airway obstruction [5]. As a consequence, clinical presentation may be less dramatic, increasing the likelihood of delayed recognition. In our study, a subset of patients did not provide a clear or timely history of aspiration, and this was significantly associated with prolonged disease duration. These findings suggest that delayed or absent reporting of aspiration history is an important factor contributing to delayed diagnosis, particularly in school-aged children who may conceal the event.

From a diagnostic perspective, chest CT identified and localized the foreign body in all patients who underwent CT in this cohort and also allowed assessment of secondary pulmonary changes. However, these findings should be interpreted cautiously. This study was not designed to assess the diagnostic accuracy of CT, did not include a direct comparison with chest radiography, and did not collect standardized data on whether CT findings changed the indication for bronchoscopy, instrument selection, bronchoscopic approach, operative time, or outcomes. Therefore, CT should not be interpreted as a routine requirement for all children with suspected airway foreign body aspiration.

In clinical practice, urgent bronchoscopy should not be delayed in children with airway instability or a definite aspiration history with significant respiratory symptoms. For clinically stable children, CT may be considered selectively, particularly when the aspiration history is unclear or delayed, symptoms are atypical, radiographic findings are inconclusive, or more precise localization is needed for procedural planning. The potential benefit of CT should be balanced against radiation exposure, especially in children.

Bronchoscopy remains the cornerstone of management for pediatric tracheobronchial foreign bodies [8, 9]. In our series, rigid bronchoscopy achieved a 100% technical success rate, with flexible bronchoscopy serving as a useful adjunct in selected cases. The hollow structure of pen caps and pen nibs allows intraluminal grasping, which may provide a stable retrieval approach. However, impacted objects or unfavorable orientation may still present technical challenges and require individualized strategies.

Although no life-threatening complications were observed, clinically relevant perioperative events did occur, including transient hemoptysis, airway edema, and pneumothorax in isolated cases. These findings underscore the importance of careful airway management, appropriate anesthetic support, and multidisciplinary coordination during foreign body removal [10].

This study has several limitations. First, its retrospective single-center design and relatively small sample size may limit generalizability. Second, although CT was performed in most patients, we did not perform a comparative analysis of CT versus chest radiography, and therefore the diagnostic performance of CT could not be formally assessed [11, 12]. We also did not collect standardized data on whether CT findings changed the decision to perform bronchoscopy, choice of instruments, bronchoscopic approach, operative time, or clinical outcomes. Third, bronchoscopic images were not consistently available, which may limit the educational value of the study. Finally, long-term follow-up was limited, preventing evaluation of delayed complications [13, 14].

Despite these limitations, this study adds focused clinical information on a specific subgroup that is less well described than food-related airway foreign bodies. The contribution of this series lies not in proposing a new bronchoscopic intervention, but in characterizing the clinical and procedural implications of pen caps and pen nibs, particularly their hollow or tubular structure, occurrence in school-aged children, potential for delayed recognition when the aspiration history is absent or concealed, and practical retrieval considerations during bronchoscopy.

## Conclusions

Stationery-related tracheobronchial foreign body aspiration predominantly affects school-aged boys and presents unique diagnostic challenges. The hollow structure of these objects and delayed or absent reporting of aspiration history may contribute to delayed diagnosis and prolonged disease duration. CT may be considered as a selective adjunct for localization and procedural planning in clinically stable children, particularly when the history is unclear or presentation is atypical, while rigid bronchoscopy remains the primary diagnostic and therapeutic modality.

## Supplementary Information

Supplementary Material 1.

## Data Availability

The datasets used and or analyzed during the current study are available from the corresponding author on reasonable request.
