# Supplementary material for: Tracheobronchial pen cap and pen nib foreign body aspiration in 32 children: clinical features, CT findings, and bronchoscopic management
Source: BMC Pediatr. 2026 Jul 30;26:1066. doi: 10.1186/s12887-026-07395-0 (PMC13711882; doi:10.1186/s12887-026-07395-0)
Supplement: Supplementary file 1 [file 12887_2026_7395_MOESM1_ESM.docx]

**Title: Supplementary Case Descriptions for “Tracheobronchial aspiration of pen caps and pen nibs in children”**
This supplementary file provides detailed descriptions of three representative cases illustrating technical challenges and management strategies in tracheobronchial foreign body removal.

# Case 1

A 4-year-old boy presented with cough, wheezing, and dyspnea for 5 hours following accidental aspiration of a plastic pen tip. Chest CT confirmed a tracheal foreign body. Emergency rigid bronchoscopy was performed under combined intravenous–inhalation general anesthesia. Due to the lack of specialized retrieval instruments, conventional alligator forceps were used.
The foreign body could not be safely extracted through the glottis during the initial attempt, and there was concern for airway edema. The procedure was therefore temporarily halted after repositioning the foreign body into the right main bronchus. Following medical treatment to reduce airway edema, a second bronchoscopy was performed the next day.
During the second procedure, the direction of the pen tip was adjusted at the carina. The jaws of the alligator forceps were inserted into the hollow lumen of the pen tip to achieve a secure grasp. The foreign body was successfully removed together with the bronchoscope. The patient recovered uneventfully and was discharged after standard postoperative care.

# Case 2

A 10-year-old boy presented with cough and wheezing for 3 hours after aspiration of a cylindrical pen cap. Rigid bronchoscopy revealed that the pen cap was impacted at the base of the right middle bronchus. Multiple attempts (five attempts) at removal using rigid forceps were unsuccessful, and the procedure lasted approximately 3 hours.
Due to the difficulty of extraction and lack of flexible bronchoscopy capability within the department, a pulmonology consultation was obtained. After placement of an endotracheal tube, a flexible bronchoscope was introduced through the tube. Under improved visualization, flexible alligator forceps were used to grasp the solid end of the pen cap, allowing successful removal together with the endotracheal tube.
Postoperatively, the patient developed airway and glottic edema and required short-term intensive care monitoring. Mild hemoptysis was also observed. After supportive management, the patient recovered and was discharged without further complications.

# Case 3

A 10-year-old boy presented with persistent cough without a reported history of foreign body aspiration. A tracheal foreign body was identified on CT at a local hospital, and an initial attempt at removal using flexible bronchoscopy was unsuccessful. The patient subsequently developed severe dyspnea and was transferred to our center.
On admission, no pneumothorax was detected; however, a subsequent chest radiograph revealed significant pneumothorax. Emergency thoracic decompression was performed, followed by rigid bronchoscopy under general anesthesia. The foreign body was carefully rotated and removed using alligator forceps, and a chest tube was placed.
The patient’s condition stabilized rapidly. He recovered well after chest tube removal and was discharged without further complications.

These cases highlight key technical considerations in the management of pen cap and pen nib aspiration, including the importance of appropriate instrument selection, procedural flexibility, and multidisciplinary coordination in complex situations.
